# Circadian Clock Gene *Per2* Is Not Necessary for the Photoperiodic Response in Mice

**DOI:** 10.1371/journal.pone.0058482

**Published:** 2013-03-07

**Authors:** Keisuke Ikegami, Masayuki Iigo, Takashi Yoshimura

**Affiliations:** 1 Laboratory of Animal Physiology, Graduate School of Bioagricultural Sciences, Nagoya University, Nagoya, Japan; 2 Department of Applied Biochemistry, Utsunomiya University, Utsunomiya, Japan; 3 Avian Bioscience Research Center, Graduate School of Bioagricultural Sciences, Nagoya University, Nagoya, Japan; 4 Institute of Transformative Bio-molecules (WPI-ITbM), Nagoya University, Nagoya, Japan; Pennsylvania State University, United States of America

## Abstract

In mammals, light information received by the eyes is transmitted to the pineal gland via the circadian pacemaker, i.e., the suprachiasmatic nucleus (SCN). Melatonin secreted by the pineal gland at night decodes night length and regulates seasonal physiology and behavior. Melatonin regulates the expression of the β-subunit of thyroid-stimulating hormone (TSH; *Tshb*) in the pars tuberalis (PT) of the pituitary gland. Long day-induced PT TSH acts on ependymal cells in the mediobasal hypothalamus to induce the expression of type 2 deiodinase (Dio2) and reduce type 3 deiodinase (Dio3) that are thyroid hormone-activating and hormone-inactivating enzymes, respectively. The long day-activated thyroid hormone T_3_ regulates seasonal gonadotropin-releasing hormone secretion. It is well established that the circadian clock is involved in the regulation of photoperiodism. However, the involvement of the circadian clock gene in photoperiodism regulation remains unclear. Although mice are generally considered non-seasonal animals, it was recently demonstrated that mice are a good model for the study of photoperiodism. In the present study, therefore, we examined the effect of changing day length in *Per2* deletion mutant mice that show shorter wheel-running rhythms under constant darkness followed by arhythmicity. Although the amplitude of clock gene (*Per1*, *Cry1*) expression was greatly attenuated in the SCN, the expression profile of arylalkylamine *N*-acetyltransferase, a rate-limiting melatonin synthesis enzyme, was unaffected in the pineal gland, and robust photoperiodic responses of the *Tshb*, *Dio2,* and *Dio3* genes were observed. These results suggested that the *Per2* clock gene is not necessary for the photoperiodic response in mice.

## Introduction

Many organisms living outside of the tropical zone adapt their physiology and behavior in response to seasonal environmental changes. Such adaptations include seasonal reproduction, migration, molting, and hibernation. Among various seasonal time cues (e.g., day length [photoperiod], ambient temperature, and rainfall [1]–[5]), most organisms use the photoperiod as a calendar because it is the most accurate natural predictor of the annual phase [6]–[9]. Therefore, this phenomenon is called photoperiodism.

In mammals, the eyes are the only photoreceptive organs, and light information received by them is transmitted to the pineal gland via the suprachiasmatic nucleus (SCN), the master circadian pacemaker [10], [11]. Melatonin is synthesized in the pineal gland from serotonin by a rate-limiting enzyme, i.e., arylalkylamine *N*-acetyltransferase (Aanat), during the night and decodes the night length. Melatonin plays a deterministic role in regulating seasonal reproduction in mammals. For example, pinealectomy prevents seasonal reproduction, whereas melatonin administration mimics the effect of short days on reproductive function [10]–[12]. Although the mode of action of melatonin was a mystery for a long time, recent studies have uncovered its downstream pathway that regulates seasonal reproduction. Dense melatonin receptors are expressed by thyrotrophs within the pars tuberalis (PT) of the pituitary gland [13], [14]. Melatonin suppresses expression of the β-subunit of thyroid-stimulating hormone (TSH; *Tshb*) in the PT through the MT1 melatonin receptor [15], [16].

The duration of melatonin secretion is shorter under long day conditions than under short day conditions, meaning that melatonin cannot suppress *Tshb* in the PT under long day conditions. Long day-induced PT-derived TSH acts as the “springtime hormone” to alert the mediobasal hypothalamus (MBH) of spring in quail, sheep, and mice [17]–[19]. PT TSH acts on the TSH receptor located within the ependymal cells (ECs) lining the ventrolateral walls of the third ventricle within the MBH and induces expression of type 2 deiodinase (*Dio2*) and suppresses expression of type 3 deiodinase (*Dio3*) genes [17], [19]. Dio2 is a thyroid hormone-activating enzyme that converts prohormone thyroxine (T_4_) to bioactive triiodothyronine (T_3_), while Dio3 is a thyroid hormone-inactivating enzyme that metabolizes T_4_ and T_3_ to inactive rT_3_ and T_2_, respectively [20], [21]. Long day-induced T_3_ within the MBH appears to regulate seasonal gonadotropin-releasing hormone secretion from the hypothalamus to the pituitary gland to regulate seasonal reproduction [22]. Recent studies have demonstrated that *Eya3* may regulate *Tshb* expression with circadian clock-controlled *TEF* and *HLF* genes [23], [24].

It is well established that the circadian clock is involved in the regulation of photoperiodism in various vertebrates including fish [25], reptiles [26], birds [27], [28], and mammals [29], [30]. It has been proposed that the photoperiod is encoded at the neuronal network level of the SCN [31], [32]. It has also been suggested that circadian clock genes within the SCN are sensitive to seasonal time, leading to the encoding and decoding of seasonal information [33]–[37]. Pittendrigh and Minis proposed the “internal coincidence model” for photoperiodic time measurement [38]. This model assumes the existence of 2 internal oscillators that change their phase relationship under changing photoperiods. High-amplitude 24-h cycles of circadian clock gene expression were observed in the ovine PT [39]. *Per* expression peaked during the day, whereas *Cry* expression peaked early at night. The phase relationship between the morning *Per* peaks and the evening *Cry* peak changed among photoperiods, and the Per–Cry protein–protein interaction (i.e., internal coincidence timer) is proposed to provide a potential mechanism for generating the photoperiodic response [39].

Although mice are generally considered non-seasonal breeders and their testicles do not show seasonal size changes, they were recently shown to be an excellent model for the study of photoperiodism for observing gene expression as a marker at the hypothalamo-hypophyseal level [19]. Most inbred strains of mice (e.g., C57BL, 129, DBA, BALB) cannot produce melatonin because they genetically lack Aanat enzyme activity [40]. Therefore, these melatonin-deficient mice do not respond to photoperiodic changes. However, they do show clear photoperiodic responses to melatonin administration at the gene expression level (i.e., downregulation of *Tshb* and *Dio2,* upregulation of *Dio3*). In contrast, melatonin-proficient strains (e.g., CBA and C3H) show clear photoperiodic responses of *Tshb*, *Dio2,* and *Dio3* to changing day lengths, although the gene switches of *Tshb*, *Dio2*, and *Dio3* are not sufficient to cause photoperiodic gonadal responses.

Although the circadian clock is involved in the regulation of photoperiodism, the involvement of the circadian clock gene and the internal coincidence timer within the PT in the photoperiodic responses of *Tshb*, *Dio2,* and *Dio3* remains unclear. Among the various clock genes, *Per2* appears to be one of the most important genes because *Per2* mutant mice show arrhythmic locomotor activity under constant darkness, whereas most of the clock gene null mice do not show circadian rhythms [41]–[46]. The *Per2* gene is also proposed to be a component of the internal coincidence timer [39]. To test whether the circadian clock gene *Per2* is involved in photoperiodic response, we generated melatonin-proficient *Per2* deletion mutant mice using the speed congenic method and examined the temporal expression profiles of *Per1, Cry1,* and *Cry2* in the SCN, the pineal gland, and the PT. We also examined *Tshb*, *Dio2*, and *Dio3*, key genes in the regulation of the photoperiodic response, under short day and long day conditions.

## Materials and Methods

### Generation of Melatonin-proficient *Per2* Mutant Mice

Melatonin-deficient *Per2* deletion mutant mice (B6.129S7-*Per2 ^tm1Brd^*/J) were obtained from the Jackson Laboratory (Bar Harbor, ME, USA). Genotyping of the *Per2* locus of the progeny was carried out using polymerase chain reaction (PCR) analysis of the genomic DNA by using a separated PCR protocol (version 1; Jackson Laboratory). The primers used included the following: forward 1, 5′-cttgggtggagaggctattc-3′; forward 2, 5′-cattgggaggcacaagtcag-3′; reverse 1, 5′-aggtgagatgacaggagatc-3′; and reverse 2, 5′-gagctgcgaacacatcctca-3′. These mutant mice were backcrossed for 6 generations with the melatonin-proficient CBA/NSlc (Nihon SLC, Shizuoka, Japan) mice. Male wild-type (*Per2*+/+) and homozygote (*Per2* m/m) individuals, which were the most enriched for CBA-type microsatellite markers, were selected using the speed congenic method (Table S1). This study was approved by the Animal Experiment Committee of Nagoya University.

### Animals and Treatment

Male 4-week-old N7F5 (CBA background *Per2*+/+ and *Per2* m/m) mice were kept under short-day conditions (8 h of light, 16 h of darkness; 8L16D) for 3 weeks in light-tight boxes placed in a room at a temperature of 23±1°C. Food and water were provided *ad libitum*. At the age of 7 weeks, the mice were divided into 2 groups [19]: the first group was transferred into long-day conditions (16L8D) in which the light onset was advanced by 8 h [24], while the second group was maintained under short-day conditions (8L16D) for 2 weeks. Brains of the 9-week-old mice were collected every 4 h (n = 4–5) under 16L8D or 8L16D, respectively.

### Recording of Circadian Locomotor Activity

Mice were kept in individual cages equipped with running wheels that were placed in a light-tight box as previously described [47]. Wheel-running activity rhythm was continuously recorded by a computer system (The Chronobiology Kit; Stanford Software System, Stanford, CA, USA).

### *In situ* Hybridization

*In situ* hybridization was conducted as previously described [48]. Sense and antisense 45-mer oligonucleotide probes for each specific gene were labeled with [^33^P] dATP (NEN Life Science Products, Boston, MA, USA) using terminal deoxyribonucleotidyl transferase (Invitrogen Life Technologies, Inc.). Coronal sections (20-µm thick) of the SCN, the pineal gland, and the MBH were prepared using a cryostat (Leica Microsystems, Inc.). Hybridization was carried out overnight at 42°C. Two high-stringency post-hybridization washes were performed at 55°C. The sections were air dried and apposed to BioMax MR Film (Eastman Kodak) for 2 weeks. The following antisense gene-specific probes were used:

*Per1*: 5′-gtccctggtgctttaccagatgcacatccttacagatctgctgga-3′.

*Cry1*: 5′-cgcgcccagcgtccggaggacacgcataccttccagggcctgaga-3′.

*Cry2*: 5′-aagcactgcaggacagccacatccagctgcctgcattcacactga-3′.

*Aanat*: 5′-gtcagcgactcctgagtaagtctctccttgtcccacagcgagcca-3′.

*Eya3*: 5′-ggaggtagtccgtaagtttgggttgcctgagggtagacagcgtag-3′.

*Tshb*: 5′-gccattgatatcccgtgtcatacaatacccagcacagatggtggtg-3′.

*Dio2*: 5′-tgcttgagcagaatgaccgagtcatagagcgccaggaagaggcag-3′.

*Dio3*: 5′-ctggtaaccgtcggggccacggcctccctggtacatgatggtgcc-3′.

### Analysis of Gene Expression Rhythmicity

When statistically significant differences were observed by one-way analysis of variance (ANOVA), cosinor curve fitting was performed as previously described [49]. Genes with values of R^2^>0.2 were classified as rhythmic genes.

## Results

### Generation of Melatonin-proficient *Per2* Deletion Mutant Mice

**Figure 1:**
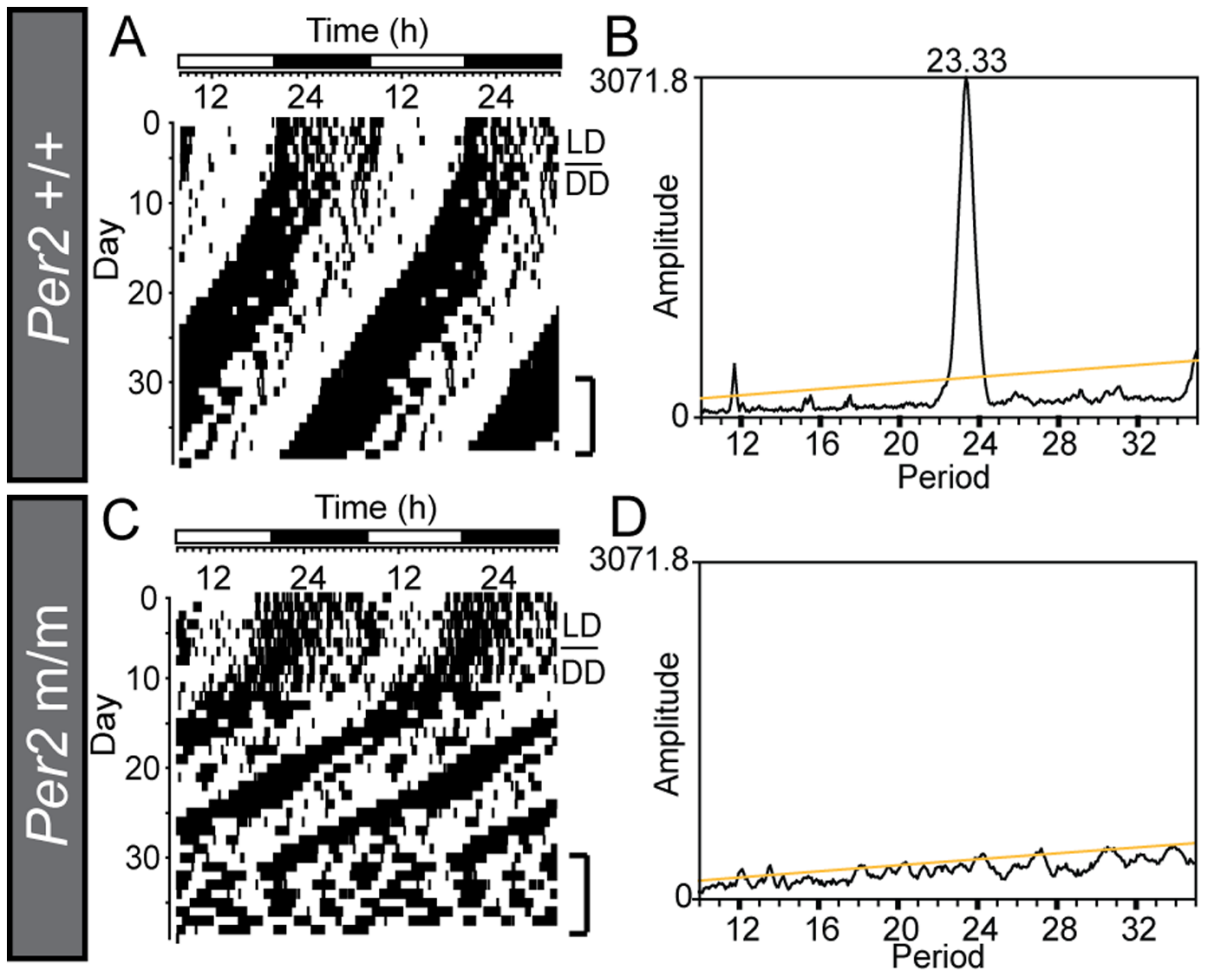
Wheel-running activity rhythms of melatonin-proficient wild-type and *Per2* deletion mutant mice. (A, C) Locomotor activity of melatonin-proficient wild-type (*Per2*+/+) and *Per2* deletion mutant (*Per2* m/m) mice in a running wheel and (B, D) their periodogram analyses under constant darkness. (A, C) The bar over the records indicates the light-dark cycles. The mice were transferred from 12L12D to constant darkness (DD). The *Per2* mutant mice showed shorter free-running rhythms followed by arhythmicity. Periodogram analyses were performed during the last 7 days. The peak above the diagonal line (<0.1%) indicates the significant circadian period (B).

We generated melatonin-proficient *Per2* deletion mutant mice by using the speed congenic method with microsatellite markers. When we examined the wheel-running activity rhythms, the *Per2* mutant mice (*Per2* m/m) displayed a shorter free-running period followed by circadian rhythm loss in constant darkness (Figures 1C, 1D), while the wild-type mice (*Per2*+/+) showed clear circadian rhythms (Figures 1A, 1B).

### Rhythmic Expression of Clock Genes is Attenuated in the SCN of *Per2* Mutant Mice

**Figure 2:**
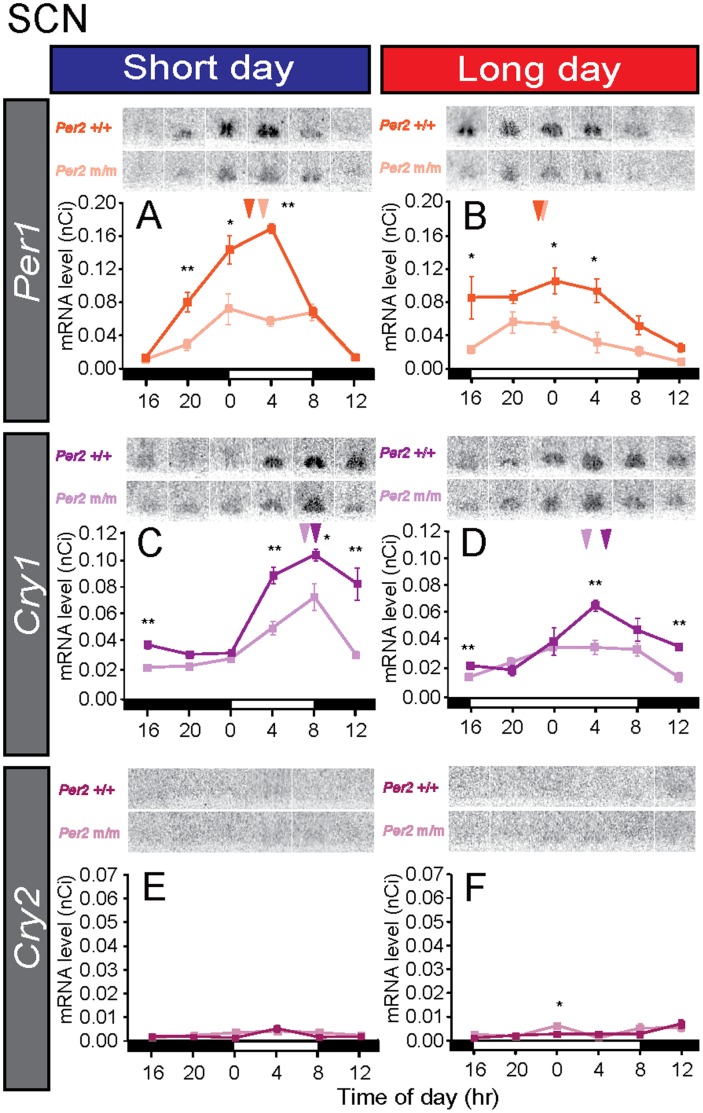
Expression analysis of clock genes in the suprachiasmatic nucleus (SCN) under short/long day conditions. Temporal changes in *Per1* (A, B), *Cry1* (C, D), and *Cry2* (E, F) expressions in the SCN of the wild-type and *Per2* mutant mice under short-day and long-day conditions. The dark and light lines in each graph represent the data of the wild-type and the *Per2* mutant mice, respectively. The bars at the bottom of each graph represent the light conditions. Representative autoradiograms of the SCN are also shown. *P<0.05, **P<0.01 *Per2*+/+ vs. *Per2* m/m at the same time point (Student’s *t*-test), mean ± SEM (n = 4–5). The arrowhead indicates the peak phase determined using cosinor curve fitting.

We examined the expression of the circadian clock genes *Per1*, *Cry1,* and *Cry2* in the SCN in wild-type and *Per2* mutant mice. In the wild-type mice, high *Per1* and *Cry1* expression levels during the light phase and around the light offset, respectively, were observed under both short-day and long-day conditions (Figures 2A–2D). Although the phases of these rhythmic expressions of *Per1* and *Cry1* were not altered, amplitude was greatly attenuated in the *Per2* mutant mice (two-way ANOVA, *P<*0.01) (Figures 2A–2D). The expression level of *Cry2* was very low in the SCN in both the wild-type and *Per2* mutant mice (Figures 2E, 2F). This result was consistent with those of an earlier report [50].

### *Per2* is not Essential for Pineal Clock Function

**Figure 3:**
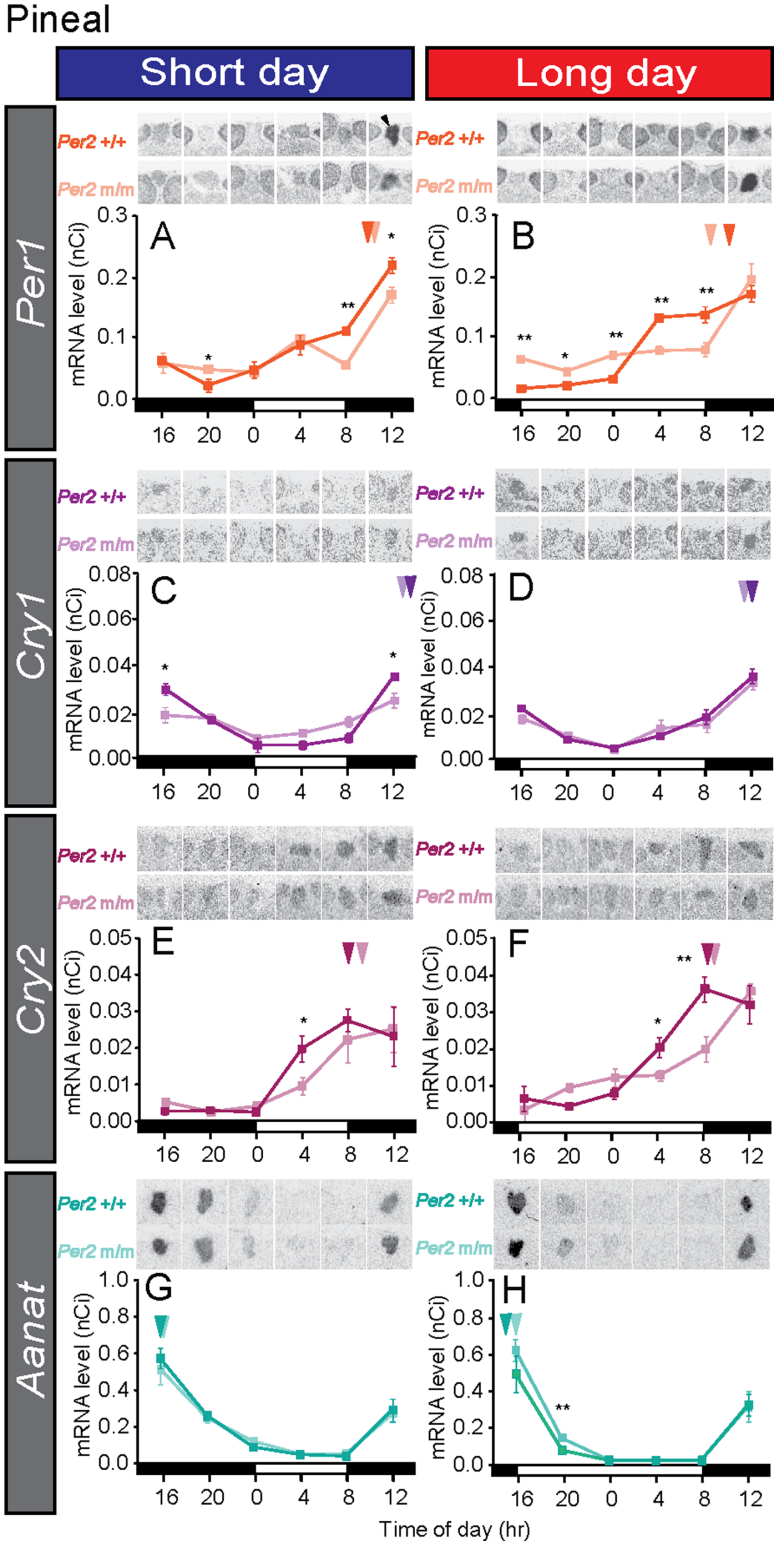
Temporal expression profiles of the circadian clock genes and arylalkylamine *N*-acetyltransferase (*Aanat*) gene expressions in the pineal gland. Temporal gene expressions of *Per1* (A, B), *Cry1* (C, D), *Cry2* (E, F), and *Aanat* (G, H) in the pineal gland under short-day and long-day conditions. The bars at the bottom of each graph represent the light conditions. Representative autoradiograms of the pineal gland (A, arrowhead) are also shown. *P<0.05, **P<0.01 *Per2*+/+ vs. *Per2* m/m at the same time point (Student’s *t*-test), mean ± SEM (n = 4–5). The arrowhead indicates the peak phase determined using cosinor curve fitting.

**Figure 4:**
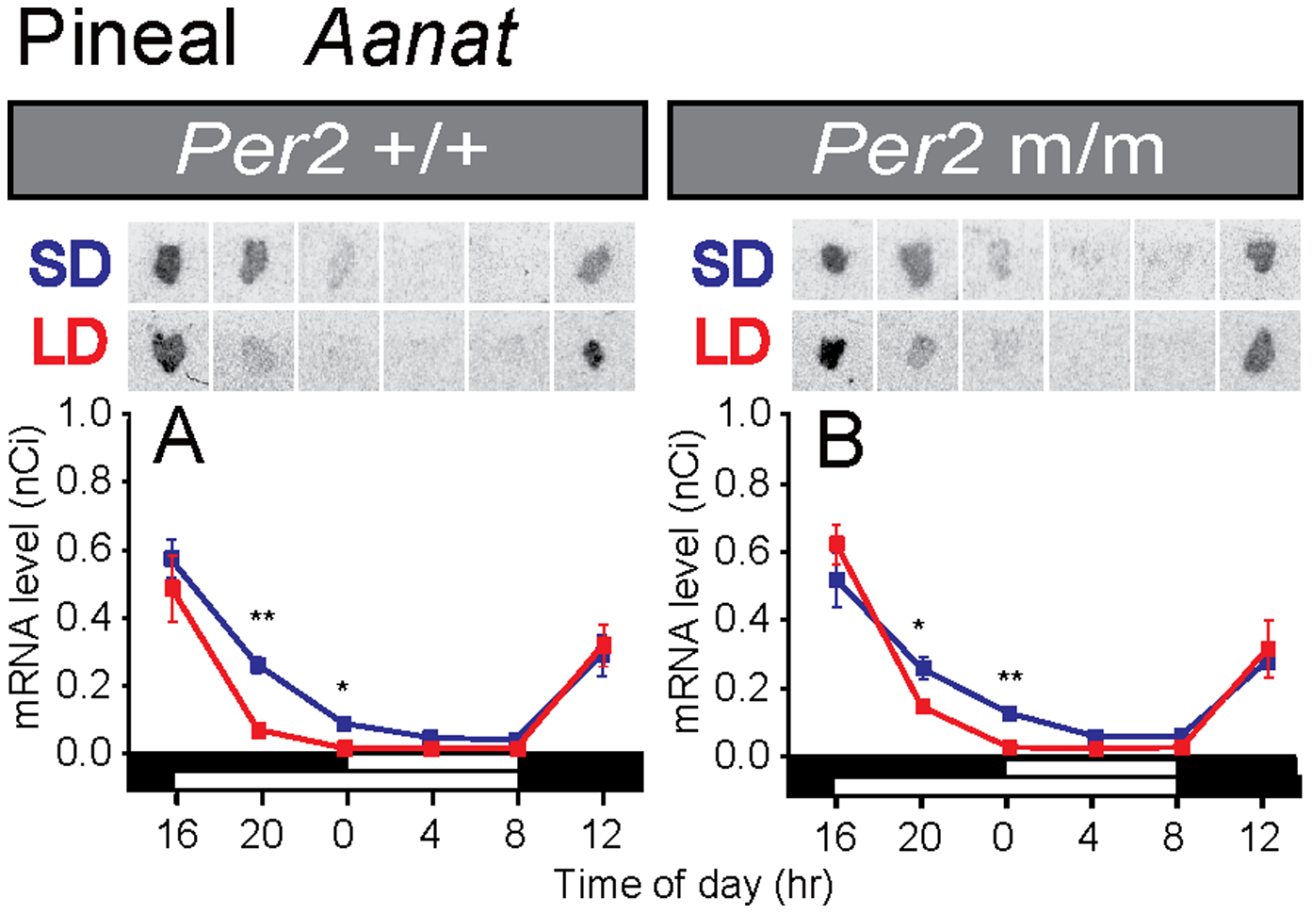
Temporal expression profiles of the *Aanat* gene in wild-type (A) and *Per2* mutant mice (B) under short-day and long-day conditions. Data were replotted from Figure 3. The bars at the bottom of each graph represent the short-day (SD; blue) and long-day (LD; red) conditions. Representative autoradiograms of the pineal gland are also shown. *P<0.05, **P<0.01 LD vs. SD at the same time point (Student’s *t*-test), mean ± SEM (n = 4–5).

We next examined the expression of *Per1*, *Cry1, Cry2,* and *Aanat*–a rate-limiting enzyme–for melatonin synthesis in the pineal gland in wild-type and *Per2* mutant mice. Although statistically significant differences were observed between wild-type and *Per2* mutant mice at some time points (Student’s *t*-test, *P*<0.05), clear rhythmicity also was observed in the *Per2* mutant mice (Figure 3) (one-way ANOVA, *P*<0.01), suggesting that *Per2* gene deletion has little effect on pineal clock function. The duration of *Aanat* expression was shorter under long-day conditions than under short-day conditions in both strains (Figure 4). We measured the serum melatonin levels in *Per2*+/+ and *Per2* mutant mice using radioimmunoassay. However, since serum melatonin levels are very low in mice, we could not obtain reliable data regarding serum melatonin rhythms in the present study.

### No Clear Internal Coincidence Timer in the PT in Mice

**Figure 5:**
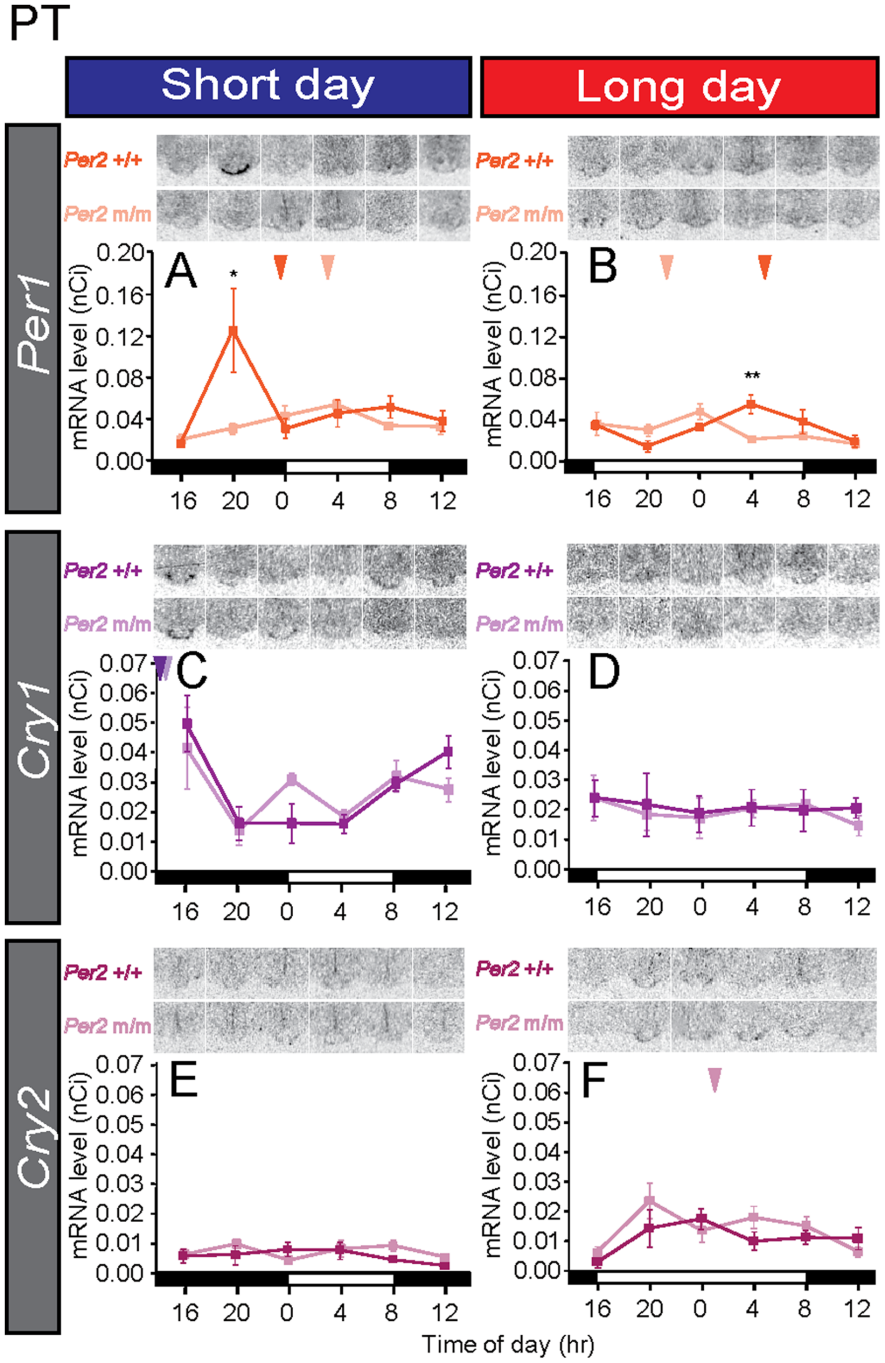
No clear internal coincidence timer in the mouse pars tuberalis (PT). Temporal expression profiles of the circadian clock genes (*Per1*, *Cry1,* and *Cry2*) in the PT of wild-type and *Per2* mutant mice. The bars at the bottom of each graph represent the light conditions. The dark and light lines in each graph represent the data of the wild-type and the *Per2* mutant mice, respectively. *P<0.05, **P<0.01 *Per2*+/+ vs. *Per2* m/m at the same time point (Student’s *t*-test), mean ± SEM (n = 4–5). The arrowhead indicates the peak phase determined using cosinor curve fitting.

Clock gene expression is melatonin-dependent in the mammalian PT [51]–[53]. Under short-day conditions, strong *Per1* expression late at night and *Cry1* expression at midnight were observed in the wild-type mice (Figures 5A, 5C). This result was consistent with that of an earlier report using C3H mice kept under 12L12D conditions [51]. In contrast, the *Per1* and *Cry1* expression rhythmicities were not robust under long-day conditions in the wild-type mice (Figures 5B, 5D) (one-way ANOVA, *Per1*: *P*<0.05; *Cry1*: *P*>0.05). Although high *Cry1* expression levels were also observed in the *Per2* mutant mice at midnight under the short-day conditions (Figure 5C) (one-way ANOVA, *P*<0.05), its rhythmicity was not clear under long-day conditions (Figure 5D) (one-way ANOVA, *P*>0.05). Although low-amplitude rhythmicity in *Per1* expression was observed under both short-day and long-day conditions in the *Per2* mutant mice (one-way ANOVA, *P*<0.05), its peak phase was not consistent with that in the wild-type mice (Figures 5A, 5B). The *Cry2* expression level was very low and robust rhythmicity was not observed in the PT of wild-type or *Per2* mutant mice (Figures 5E, 5F) (*Per2*+/+ long day and short day, *Per2* m/m short day: one-way ANOVA, *P*>0.05; *Per2* m/m long day: one-way ANOVA, *P*<0.05), a finding consistent with those of previous in rodents [54], [55].

### *Per2* Mutant Mice Show Robust Photoperiodic Responses at the Gene Expression Level

**Figure 6:**
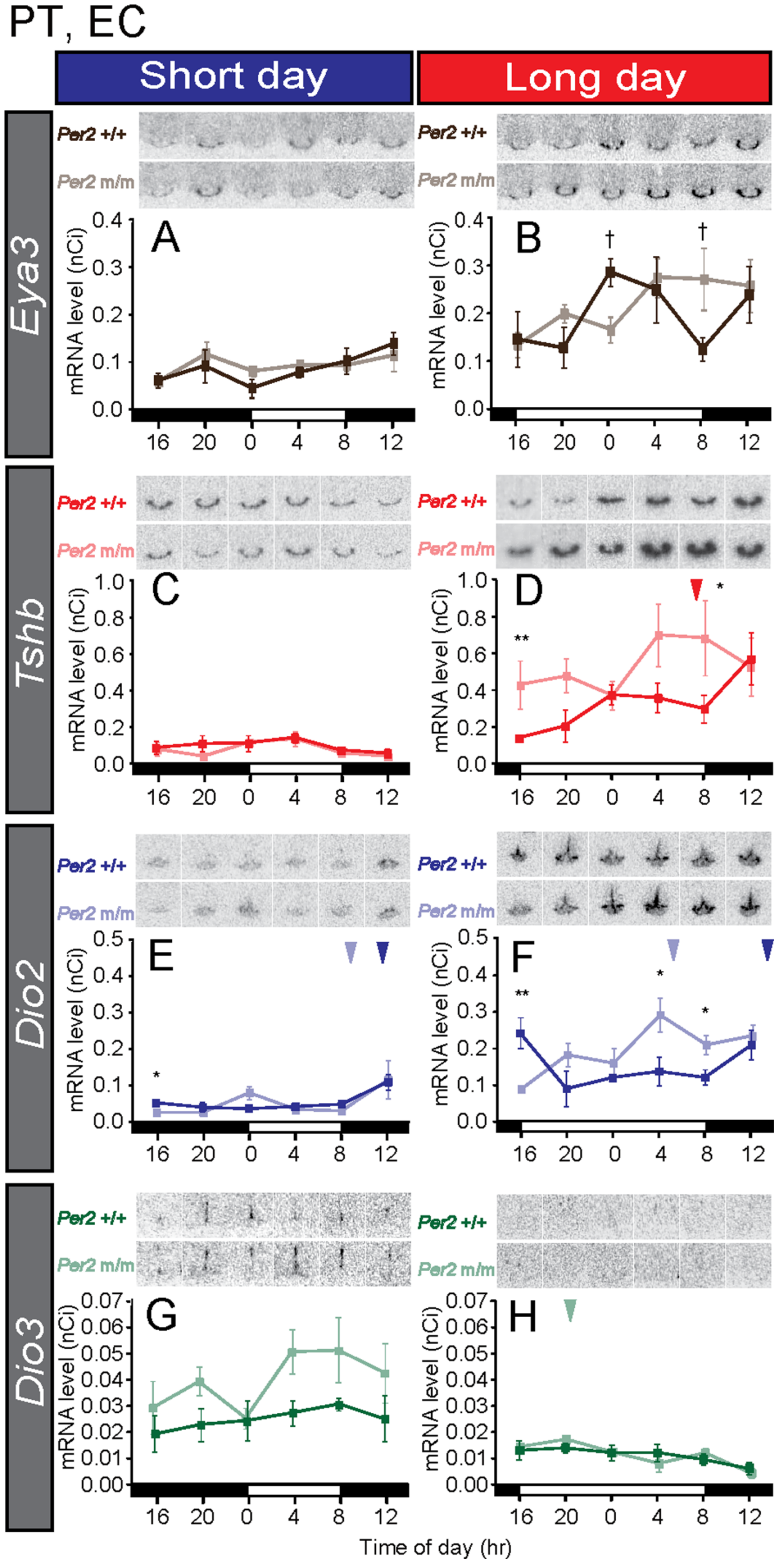
The photoperiodic response of key genes regulating seasonal reproduction in wild-type and *Per2* mutant mice. The bars at the bottom of each graph represent the light conditions. The dark and light lines in each graph represent the data for the wild-type and the *Per2* mutant mice, respectively. *P<0.05, **P<0.01 *Per2*+/+ vs. *Per2* m/m at the same time point (Student’s *t*-test), mean ± SEM (n = 4–5). The arrowhead indicates the peak phase determined using cosinor curve fitting.

The ability of mice to show a photoperiodic response was tested after 2 weeks of either short or long days. When we examined the expression of *Eya3* and *Tshb* in the PT and *Dio2* and *Dio3* in the ECs, long-day induction of *Eya3*, *Tshb,* and *Dio2* and suppression of *Dio3* expression were observed in both wild-type and *Per2* mutant mice (Figure 6) (two-way ANOVA, *P<*0.01, short day vs. long day). Interestingly, *Tshb* and *Dio2* expression induction under long-day conditions and *Dio3* expression under short-day conditions were more robust in *Per2* mutant mice than in wild-type mice (Figures 6D, 6F, 6G) (two-way ANOVA, *P<*0.01, wild-type vs. *Per2* mutant). Statistical analysis demonstrated rhythmic expression of the *Dio2* gene in both wild-type and *Per2* mutant mice under short- and long-day conditions (Figure 6E, 6F). However, its peak phase was different between the 2 genotypes under long-day conditions (Figure 6F). The temporal expression profiles of *Eya3* and *Tshb* were also different between the 2 genotypes under long-day conditions (Figure 6B, 6D).

## Discussion

It is well established that the circadian clock is involved in photoperiodic time measurement in various organisms [25]–[30]; however, the involvement of the circadian clock genes in the photoperiodic response has remained unclear; to elucidate the involvement, we decided to use the mouse model because gene-targeting techniques are unavailable in other photoperiodic mammals such as hamsters and sheep. Although the original *Per2* deletion mutant mice have shown arhythmicity under constant darkness [42], they were unable to produce melatonin. Therefore, we first generated melatonin-proficient *Per2* mutant mice using the speed congenic method. Although a different genetic background sometimes alters the circadian phenotype [56], the wheel-running activity rhythms of melatonin-proficient *Per2* mutant mice were similar to those of original melatonin-deficient *Per2* mutant mice (Figure 1). When we examined the temporal expression profiles of *Per1* and *Cry1* expression within the SCN and found that the gene expression amplitude was greatly attenuated (Figure 2). This result is consistent with the results seen in the original melatonin-deficient *Per2* mutant mice [42]. Recent studies have suggested that core circadian clock elements within the SCN encode and decode seasonal information [33]–[37]. Although clock gene expression amplitude was greatly attenuated in the *Per2* mutant mice, phase relationships of *Per1*/*Cry1* appeared to be conserved (Figure 2). Unexpectedly, the temporal expression profiles of *Per1*, *Cry1,* and *Cry2* did not differ greatly in the pineal gland between the *Per2* mutant and wild-type mice (Figure 3). In addition, the expression profile of *Aanat* was almost identical between the 2 genotypes, suggesting that *Per2* is not very important for pineal clock function in mice, at least in the presence of a light-dark cycle. This finding was in marked contrast with the findings in melatonin-proficient C3H *Per1* deficient mice that showed altered *Aanat* expression and melatonin synthesis [57]. Therefore, we suggest that *Per1* plays a more important role than *Per2* in the pineal gland. Although we found clear rhythmicity of the clock genes and *Aanat* in the pineal gland of *Per2* mutant mice, it is possible that the photoperiodic response in *Per2* mutant mice in fact reflects the acute suppression of *Aanat* by light. To test this possibility, it is important to measure *Aanat* expression under constant dark conditions. It is also possible to speculate that the conserved phase relationship between *Per1*/*Cry1* in the SCN of *Per2* mutant mice enabled the pineal gland to retain seasonal information about melatonin even though the *Per1*/*Cry1* amplitude was greatly attenuated.

When we looked at *Per1* and *Cry1* expressions in the PT, strong *Per1* expression late at night and strong *Cry1* expression at midnight were observed in wild-type mice under short-day conditions (Figure 5). Although the timings of the *Per1* and *Cry1* peaks were consistent with those in earlier reports in C3H mice [51], [58], those timings were slightly different from the results of hamsters [59], [60]. *Per* is believed to be expressed at dawn in response to declines in melatonin signal. Therefore, differences in gene expression profiles among species are probably caused by differences in temporal melatonin secretion profiles among species [61], [62]. In contrast to the short-day condition, no robust rhythmicity was observed in either *Per1* or *Cry1* expression under long-day conditions in either genotype (Figure 5). Since clock gene expression in the PT is melatonin dependent [52], [63] and the duration of *Aanat* expression was slightly shorter under long-day than under short-day conditions (Figure 4), the long-day secretion profile of melatonin appeared to be insufficient for generating rhythmic clock gene expression in the PT under long-day conditions (Figure 5). In the *Per2* mutant mice, we observed robust rhythmic *Cry1* expression under short-day conditions only and *Per1* expression rhythmicity was weak (Figure 5). The *Cry2* expression level was very low in the PT and the SCN under both short-day and long-day conditions (Figures 2, 5).

In this study, despite the lack of clear evidence of the existence of a *Per–Cry* internal coincidence timer in the PT, we found robust photoperiodic responses of the *Eya3* and *Tshb* genes in the PT and of the *Dio2* and *Dio3* genes in the ECs (Figure 6). These results were in marked contrast with those in the sheep PT, which showed clear phase angle differences in the *Per* and *Cry* genes under both short-day and long-day conditions [39], suggesting that the *Per–Cry* internal coincidence timer in the PT is not necessary for the photoperiodic responses of the *Eya3*, *Tshb*, *Dio2,* and *Dio3* genes in mice. If the internal coincidence timer within the PT mediates the photoperiodic control of summer or winter physiology and is central in the regulation of photoperiodism, the internal coincidence timer could be highly conserved among various species. The differences observed in PT clock gene expression profiles among species suggest that the internal coincidence timer in the PT observed in or study is not a universal mechanism. Although we failed to find clear internal coincidence timer in the mouse PT, we found different expression profiles of the *Per1*, *Eya3*, *Tshb*, and *Dio2* genes between the 2 genotypes under long-day conditions. Different temporal expression profiles of the clock genes (*Per1*) and *Eya3* between the 2 genotypes may reflect the different temporal expression profiles of *Tshb* and *Dio2* gene expression under long-day conditions. Generation of a floxed allele of circadian clock gene driven by a PT-specific gene Cre driver line will clarify the functional significance of circadian clock in the PT in the regulation of photoperiodism.

The circadian clock gene *Per2* plays a critical role in the regulation of circadian locomotor activity rhythms. However, our present study showed that *Per2* is not necessary for photoperiodic responses in mice, at least within 2 weeks of photoperiodic exposure. Although we have previously reported clear photoperiodic responses within 2 weeks in birds and mammals [17], [19], [20], 2 weeks is a rather short period of time compared to durations that are usually used for sheep. Therefore, we cannot exclude the possibility that a study duration longer than the current one may reveal some differences between the 2 genotypes. The *tau* mutation of the circadian clock of the Syrian hamster responded to programmed systemic infusions of melatonin in a manner comparable to that observed in wild-type hamsters [64]. However, this mutation altered the photoperiodic responsiveness of the gonadal axis to melatonin signal frequency, suggesting a role for the circadian clock in the interpretation of a series of signals and the subsequent generation of a photoperiodic response [65]. Thus, the circadian clock is known to be involved in photoperiodic time measurement in various organisms [25]–[30]. However, we cannot totally exclude the possibility of the involvement of a completely different set of “clock genes” for the photoperiodic time measurement. The existence of a circadian clock mechanism that lacks a transcription–translation feedback loop was recently suggested in some studies [66], [67], but how the circadian clock measures day length (i.e., how it defines the photoinducible phase or critical photoperiod)–the heart of the photoperiodic time measurement–remains unanswered.

## Supporting Information

Table S1 **Microsatellite markers used to generate speed congenic mice.** Microsatellite loci and polymerase chain reaction annealing temperature (°C) are shown.
(TIF)
