# Supplementary figures and images for: Circadian Clock Gene Per2 Is Not Necessary for the Photoperiodic Response in Mice
Source: PLoS One. 2013 Mar 7;8(3):e58482. doi: 10.1371/journal.pone.0058482 (PMC3591342; doi:10.1371/journal.pone.0058482)

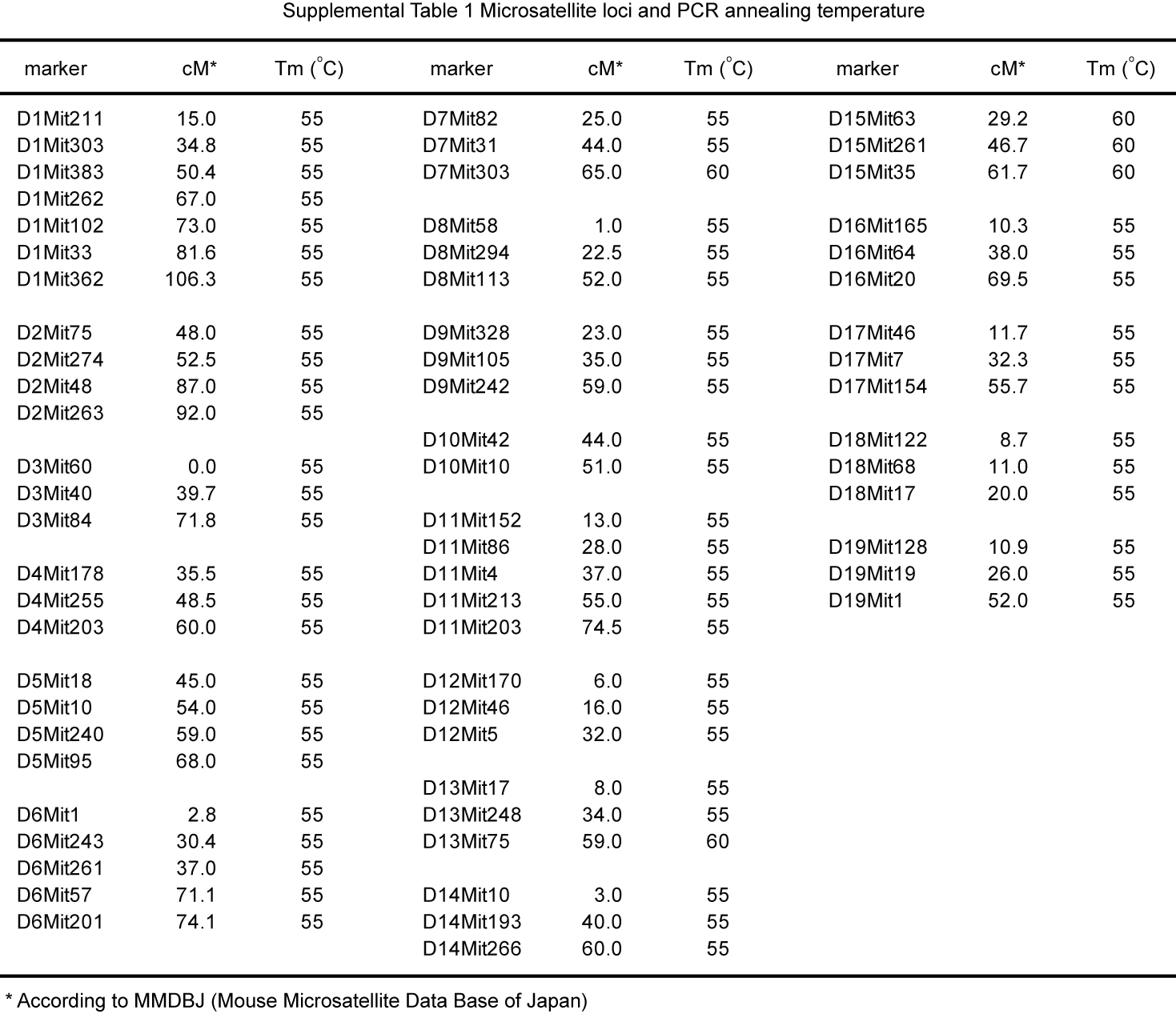

Supplement: Table S1 — Microsatellite markers used to generate speed congenic mice. Microsatellite loci and polymerase chain reaction annealing temperature (°C) are shown. (TIF) [file pone.0058482.s001.tif]
